# Editorial: Diminished Ovarian Reserve and Poor Ovarian Response: Diagnostic and Therapeutic Management

**DOI:** 10.3389/fphys.2022.827678

**Published:** 2022-07-19

**Authors:** Racca Annalisa, Stoop Dominic, Polyzos Nikolaos P

**Affiliations:** ^1^ Department of Obstetrics Gynecology and Reproductive Medicine, Dexeus University Hospital, Barcelona, Spain; ^2^ Faculty of Medicine and Health Sciences, Ghent University, Ghent, Belgium

**Keywords:** diminished ovarian reserve (DOR), ovarian stimulation and response, IVF (in vitro fertilization), ART (assisted reproductive technology), poor ovarian response (POR)

Over the last decades there has been a steep increase in the demand for ART treatment and the main reason for this is the advanced age of the couple trying to conceive. Another important aspect of the last years is the increased incidence of malignancies in young-adults and the consequent fertility preservation of cancer survivors. In contrast to what people are inclined to think, *in vitro* fertilization (IVF) treatments cannot fully compensate for age-dependent loss of fertility, as the success rate of any fertility techniques directly depend on maternal age (Mills et al., 2011). In fact, advanced maternal age (Wallace and Kelsey, 2010), and iatrogenic (ovarian surgery or gonadotoxic therapies) (Dewailly et al., 2014) or non-iatrogenic conditions (for instance the presence of genetic polymorphism at the levels of gonadotropin receptors) can reduce the ovarian reserve. Independently of the cause of diminished ovarian reserve (DOR), up to 1/3 of these patients experience a poor ovarian response (POR) to ovarian stimulation (OS) leading to cycle cancellation and a reduced chance of a live birth (Polyzos et al., 2012; Polyzos et al., 2014; La Marca et al., 2015; Polyzos and Popovic-Todorovic, 2020). The first consensus on the definition of POR (Ferraretti et al., 2011), the Bologna criteria, has been the first time this population was clinically defined; however, the most important limitation was the heterogeneity of the population included in the definition, given by grouping women with different biological characteristics and therefore prognosis (Polyzos and Drakopoulos, 2019). More recently a different grouping of these patient was proposed, based on age and ovarian sensitivity to OS, two features that may impact the prognosis (Esteves et al., 2019).

Surely POR still represent one of the most difficult subgroups of IVF patients to treat in the everyday clinical practice. Therefore, we set up this Research Topic with the aim to provide a comprehensive overview of the diagnostic and therapeutic management of patients with DOR and POR from different perspectives: definition, diagnostic and etiology of DOR, efficacy of different ART for the patient’s management; and lastly, novel and promising strategies for the treatment of DOR and POR.

As in many other situations, in IVF the capacity to predict a possible failure is crucial. With the objective to prevent a critical outcome, the first step is to define DOR and describe which test can be performed to diagnose women with DOR. Moreover, in the literature, the clinical use of ovarian reserve markers (ORMs) is based on the use of cut-off points. However, the cut-offs are very frequently arbitrary, depending on the different definitions of DOR, the different measuring methods and lastly the high heterogeneity of the population investigated. For the definition and diagnosis of DOR, Wang et al., explored the ovarian reserve tests (ORTs) and their respective values, also according to specific age cut-offs, in order to predict poor ovarian response and to personalize IVF treatment appropriately. The main result showed that age, AFC, AMH and basal FSH are predicting factors for POR, where AFC and AMH are the best, if using only a single factor as predictor. AMH has a very low intra- and inter-cycle variability, thereby offering a good quantitative and qualitative follicle marker compared to clinical and endocrine ones; it is therefore the best single predictor of POR. Along similar lines of predicting ovarian response, Wen et al., investigated the reference range and the potential value of inhibin B, a non-steroidal hormone produced by the granulosa cells with a known property of FSH suppression. The main results showed that a reduction in inhibin B reflects DOR and has a good consistency with both AMH and AFC. Bai et al. , explored the ovarian response-related risk factors. They determined the expression of growth differentiation factor-8 (GDF-8), a member of the transforming growth factor β family and known to have a crucial role in folliculogenesis, and the expression of its specific receptors in different ovarian response patients during OS. The authors concluded that aging, obesity, endometriosis, ovarian surgery, and high levels of GDF-8 are high risk factors for POR.

When looking at etiology, one of the causes of DOR is the exposure to gonadotoxic medication for oncological reasons. Chemotherapy-associated ovarian failure (COF), has been described by Mauri et al. as a disruption of ovarian function both as an endocrine gland and as a reproductive organ. The real underlying mechanism by which this happens is still not fully understood; however, it seems to be associated with either DNA damage of the premature ovarian follicle or its early activation and apoptosis, resulting in the exhaustion of the follicle reserve. As a matter of fact, due to the delay in the pregnancy wish and due to the increasing percentage of women affected by malignancies, it is of the utmost importance to give any female cancer patient the opportunity to express their pregnancy wishes after any antineoplastic treatment is completed.

The definition of a unified treatment approach for POR has not yet been outlined. Given the heterogeneity of the ovarian response in the DOR population, it is questionable whether the “one size fits all” approach should still be the main research focus, or whether more refined and personalized treatment strategies should be investigated. In this direction, Papageorgiou et al. pointed out that proper molecular testing should be performed. Regulators of follicle maturation could potentially be used as prognostic biomarkers of the response to different gonadotropin regimens. In particular, the PI3K/Akt/mTOR and Hippo pathways could be monitored, as the dynamic balance between these two opposite modulators is pivotal for proper follicle maturation. However, in the absence of defined protocols based on molecular biomarkers, current research is spread over a range of heterogeneous treatment strategies.

A first line of research compared one conventional GnRH antagonist stimulation with multiple minimal OS, demonstrating the superiority of conventional OS in terms of number of oocytes retrieved and pregnancy rates Liu et al..

A second group of studies investigated the role of androgen supplementation in DOR. Despite promising results on animals, Neves et al., by reviewing the literature on DHEA, showed that there were inconclusive results on humans, due to the large heterogeneity between the studies. Notably, Chen et al., demonstrated that a faster increase in testosterone levels, from baseline to the day after the ovulation trigger, could be associated with better pregnancy outcomes.

A third research cluster aims to stimulate follicular development by triggering paracrine signaling mechanisms with either inhibition of molecular pathways together with *in vitro* activation (IVA), mechanical fragmentation, administration of bone marrow-derived stem cells (BMDSC) as well as of platelet-rich plasma (PRP) Polonio et al.; Fàbregues et al.. Although promising, such treatments are still experimental and further research is needed before translation in a clinical setting.

Finally, there are a number of stand-alone studies, possibly pioneering new frontiers in the treatment of DOR and POR. Zhu et al., found that growth hormone (GH) administration before frozen-thawed transfer would increase oocyte quantity and quality, thus improving cycle and pregnancy outcomes. Song et al., compared traditional Chinese formula Ding-Kun Pill (DKP) supplementation versus placebo in POSEIDON group 4 women and found a higher ongoing pregnancy rate in the DKP group, though the finding is based on a subgroup analysis with small sample sizes. Yang and co-workers investigated pharmacological mechanisms through which melatonin could improve ovarian reserve: in summary, melatonin was able to show anti-aging, anti-apoptotic, endocrine, and immune system regulation Yang et al. Lastly, Christodoulaki et al., proposed germline nuclear transfer (NT) as a promising new treatment for DOR patients. NT consists in the transfer of a nuclear genome from patient oocytes to enucleated donor oocytes, thus circumventing the biochemical issues related to advanced maternal age and reduced oocyte competence.

In conclusion, DOR and POR represent one of the hardest challenges in ART. As a general recommendation, a thorough exploration of the ovarian reserve and related biomarkers should always be performed as the first step towards tailored treatment strategies. However, the success rate in this population of patients is still unacceptably low. In recent years, the need for tangible improvements have pushed forward the boundaries of research and innovation. We are still at the stage of growth and exploration; however, the impressive bulk of research makes us confident that such collective effort will inevitably lead to successful outcomes in the near future.
